# Supplementary material for: Metallic Material Selection and Prospective Surface Treatments for Proton Exchange Membrane Fuel Cell Bipolar Plates—A Review
Source: Materials (Basel). 2021 May 20;14(10):2682. doi: 10.3390/ma14102682 (PMC8161061; doi:10.3390/ma14102682)
Supplement: Supplementary file 1 [file materials-14-02682-s001.zip › materials-1184080-supplementary.pdf]

materials

Supplementary Material

Metallic Material Selection and Prospective Surface Treatments for Proton Exchange Membrane Fuel Cell Bipolar Plates—A Review

Tereza Bohackova[1], Jakub Ludvik * and Milan Kouril

Department of Metals and Corrosion Engineering, University of Chemistry and Technology Prague, Technická 5 Prague 6, 166 28 Prague, Czech Republic; jamborot@vscht.cz (T.B.); kourilm@vscht.cz (M.K.)
* Correspondence: ludvika@vscht.cz

Citation: Bohackova, T.; Ludvik, J.; Kouril, M. Metallic Material Selection and Prospective Surface Treatments for Proton Exchange Membrane Fuel Cell Bipolar Plates—A Review. *Materials* **2021**, *14*, 2682. https://doi.org/10.3390/ma14102682

Academic Editor: Jeong-Soo Sohn

Received: 30 March 2021
Accepted: 10 May 2021
Published: 20 May 2021

Publisher's Note: MDPI stays neutral with regard to jurisdictional claims in published maps and institutional affiliations.

Copyright: © 2021 by the author. Licensee MDPI, Basel, Switzerland. This article is an open access article distributed under the terms and conditions of the Creative Commons Attribution (CC BY) license (http://creativecommons.org/licenses/by/4.0/).

Table S1. Comparison of corrosion resistance and contact resistance of various uncoated steels according to literature.

Material	Electrolyte	Temperature (°C)	icorr ($\mu A/cm^2$)			ICR ($m\Omega cm^2$)	Citation
			at Ecorr	at - 0.1 V/SCE	at 0.6 V/SCE	at 140 N/cm^2	
316L<br>321<br>347	85% H_3PO_4	30-120	3.16-130 μA<br>0.13-40 μA<br>0.30-79 μA				[163]
316L<br>430	0,001 M H_2SO_4, 2 ppm F^-	25	0.00249<br>0.00328				[187]
316L	H_2SO_4, pH 1-6, 2 ppm HF	80			17.91-2.24^{D}		[34]
316L	0,5 M H_2SO_4	70	2.43^{K}, 9.15^{A}	5^{D}	-0.7^{D}		[22]
316L<br>316L elchem. polishing	1 M H_2SO_4, 2 ppm F^-	70		45^{S}	 1-10^{S}	229.2$^{\text{at 150}}$<br>5.7-23.5$^{\text{at 150}}$	[49]
316L	0,001 M H_2SO_4<br>10^{-6} M H_2SO_4<br>0,5 M H_2SO_4	70			2.85$^{S,\text{at 1.4 V/SHE}}$	67.6 vs. 149$^{\text{at 137}}$ (after 1 V/SHE)<br>9.8 vs 38.1$^{\text{at 137}}$ (after 1 V/SHE)<br>10.5 vs. 6.0$^{\text{at 137}}$ (after 1 V/SHE)	[23]
316L	0,5 M H_2SO_4	70			0.8^{S,} at$^{\text{1 V/SHE}}$	885 vs. 964 (after 1 V/SHE)	[48]
316L	1 M H_2SO_4	70	2^{K}		0.2^{S}	71$^{\text{at 200}}$	[67]
316L	1 M H_2SO_4	70	20.2^{A}, 1.9^{K}	153^{D}, 145^{S}	10^{D}, 1.1^{S}	66.4$^{\text{at 274.4}}$	[69]
316L	0.5 M H_2SO_4, 5 ppm HF	70				45$^{\text{at 200}}$	[105]
316L	0.5 M H_2SO_4, 5 ppm HF	70	47.93^{A}, 30.94^{K}				[36]
316L	0.5 M H_2SO_4, 2 ppm HF	70	8.09^{A}, 39.2^{K}	11.2^{D}, 3.59^{S}	6.24E3^{D}, 4.58^{S}	159.7 vs. 238.5^{1} and 265.3$^{2,\text{ at 150}}$	[97]
316L	H_2SO_4 (pH=3, pH=6)	80				41 vs. 278 (po 1,4 V/SHE)	[25]
316L	0.5M H_2SO_4, 2 ppm HF	80		20.8^{D}	14.2^{D}, 0.6^{S}		[74]

Material	Electrolyte	Temperature (°C)		icorr ($\mu A/cm^2$)		ICR ($m\Omega cm^2$)	Citation
			at Ecorr	at - 0.1 V/SCE	at 0.6 V/SCE	at 140 N/cm^2	
316L	0.5M H_2SO_4, 2 ppm HF	25/80	0.141 /120	-9.6E-3/14.3^{D}	3.25/8.30^{D}, 0.32/0.36^{S}	156 vs. 240^{2}	[188]
316L	0.5 M H_2SO_4, 5 ppm F^-	25/70	0.32-3.16	100-31.6$^{S, \text{ at } 70\ °C}$	16/120^{D,K} a 290^{D,A}, 1-0.32$^{S, \text{ at } 70\ °C}$		[45]
316L	0.5 M H_2SO_4, 5 ppm HF	80			11.26^{D}, 1.4^{S}	255.4$^{\text{at } 210}$	[9]
316L	0.5 M H_2SO_4, 2 ppm HF	80	50^{K}, 16.5^{A}	> 1^{S}	32.3^{D}, 50^{S}	350	[78]
316L	0.5 M H_2SO_4, 2 ppm HF	80			11.26^{D}, 1.3^{S}	370.1	[81]
316L	0.1 M H_2SO_4, 2 ppm HF	80	16.5^{A}, 35.3^{K}	50^{S}	41.4^{D}, 50^{S}	350$^{\text{at } 150}$	[189]
316L	0.5 M H_2SO_4, 5 ppm HF	70					[123]
316L	0.5 M H_2SO_4, 5 ppm HF	70		5.6-10^{S}	5.1^{S}		[61]
316L	0.5 M H_2SO_4, 5 ppm HF	70			7.98^{D}		[125]
316L	0.1 M H_2SO_4		2.92				[130]
316L	10 g Na_2SO_4 (pH=3.5), 5 ppm F^-	60		-0.13^{S}	0.013^{S}	152	[86]
316	0.5 M H_2SO_4, 2 ppm HF		10.3		1.63^{S}	158	[75]
316	0.5 M H_2SO_4, 2 ppm HF	25	10.3	-6.72 to -1.45^{S}	1.54^{S}	700.5	[53]
316	0.01 M H_2SO_4 (pH=2)	80	5.66				[38]
304	0.5 M H_2SO_4, 2 ppm HF	80	2.6			140 vs. 125$^{2, \text{ at } 240}$	[41]
304	0.5 M H_2SO_4, 2 ppm HF	80				100 vs. 150^{1}, 150 vs. 340$^{2, \text{ at } 150}$	[77]
304	3.5% NaCl	25	7.41			158 vs. 560	[117]
304	0.5 M H_2SO_4, 5 ppm HF				20^{S}	124.4$^{\text{at } 150}$	[26]
304	0.5 M H_2SO_4, 2 ppm HF	70	1.01		0.144^{S}	415.32 vs. 593.45$^{2, \text{ at } 150}$	[55]
304	not specified		1.89E3	1.87E4^{D}	1.41E5^{D}	31.78 vs. 16.32	[122]
304	0.1 M H_2SO_4 (pH=3),		10				[80]
304	0.1 M H_2SO_4, 2 ppm HF	80		122^{D}	197^{D}		[30]
304	0.05 M H_2SO_4, 2 ppm F^-	70		21.2^{D}	13.45^{D}	101 vs. 170.5^{1} and 278,3^{2}	[71]

Material	Electrolyte	Temperature (°C)	icorr ($\mu A/cm^2$)			ICR ($m\Omega cm^2$)	Citation
			**at Ecorr**	**at - 0.1 V/SCE**	**at 0.6 V/SCE**	**at 140 N/cm^2**	
304	1 M H_2SO_4, 2 ppm HF	80		34.7[D], 5.28[S]	46.9[D], 33.72[S]	430	[72]
304	0.05 M H_2SO_4, 2 ppm HF	70	21.2[A] 13.45[K]			101 vs. 170.5[1] and 278,3[2]	[91]
304	0.5 M H_2SO_4, 2 ppm HF	80	78.37[A] 319.6[K]			101 vs 183.5[1] and 285.3[2]	[88]
304	H_2SO_4 (pH=5)	70			52.9[D]	98.99 vs. 160[2, at 220]	[39]
304	0.5 M H_2SO_4, 2 ppm HF	70				116 [at 135]	[47]
Carbon steel	H_2SO_4 + 0.07 M Na_2SO_4 (pH=4), 2 ppm HF	50		5.76[D]	2.51[D]		[32]
Carbon steel 1020	0.5 M H_2SO_4	25	634			403.8	[107]
Mild steel	1 M H_2SO_4	25	5240				[190]

Explanations to the table: A = anodic conditions (H_2 bubbled), K = cathodic conditions (air/oxygen purged), D = potentiodynamic test, S = potentiostatic test, 1 = after potentiostatic test at -0.1 V vs.SCE, 2 = after potentiostatic 0.6 V vs. SCE

Table S2. Comparison of corrosion resistance and contact resistance of surface treated steels.

Material	Surface treatment	Method/technique	Electrolyte	Temperature (°C)	icorr (µA/cm²)			ICR (mΩcm²)	Citation
					at Ecorr	at - 0.1 V/SCE	at 0.6 V/SCE	at 140 N/cm²	
316L	TiN	CFUBMSIP	0.001 M H_2SO_4	70			25.4-0.08$^{S,\ at\ 1.4\text{-}0.8\ V/SHE}$	12.9 vs. 287 $^{at\ 137}$ (po 1 V/SHE)	[21]
316L	TiN (0.4/1 µm)	CFUBMSIP	0.5 M H_2SO_4	70			15/11$^{S,\ at\ 1\ V/SHE}$	167/83 vs. 329/230 (po 1 V/SHE)	[46]
	TiN+C (0.4+0.1 µm)						0.02$^{S,\ at\ 1\ V/SHE}$	3.4 vs. 4.5 (po 1 V/SHE)	
	Au (10 nm)						0.05$^{S,\ at\ 1\ V/SHE}$	2.7 vs. 3.8 (po 1 V/SHE)	
316L	AlN-TiN	Plasma focus device	1 M H_2SO_4	70	2.8-9^{K}		0.03-0.6^{S}	6-20 $^{at\ 200}$	[65]
316L	TiN	EBPVD	1 M H_2SO_4	70	4.07^{A}, 31.5^{K}	10.4^{D}, 10E4^{S}	116^{D}, 18^{S}	35.0 $^{at\ 274.4}$	[67]
	CrN				1.41^{A}, 1.31^{K}	21^{D}, 10E4^{S}	52.4^{D}, 1.1^{S}	21.8 $^{at\ 274.4}$	
	TiAlN				317^{A}, 18.6^{K}	3.96E4^{D}, 10E4^{S}	1.69E4^{D}, 10E3^{S}	7.5 $^{at\ 274.4}$	
316L	TiN (0.1-1 µm)	PVD	0.5 M H_2SO_4	70				57-12 vs. 43-314	[87]
	CrN (0.1-1 µm)							339-1583 vs. 347-2121	
	ZrN (0.1-1 µm)							1364-3788 vs. 1646-3612	
316L	CrN+CrC	Chromizing deposition (900 °C)	0.5 M H_2SO_4, 5 ppm HF	70		6.5^{S}	7.5E-2^{D}	13 $^{at\ 200}$	[103]
		Chromizing deposition (1100 °C)				32^{S}	0.35^{D}		
316L	CrN	CFUBMSIP	0.5 M H_2SO_4, 5 ppm HF	70	2.231^{A}, 1.225^{K}				[34]

Material	Surface treatment	Method/technique	Electrolyte	Temperature (°C)	icorr (μA/cm²)			ICR (mΩcm²)	Citation
					at Ecorr	at - 0.1 V/SCE	at 0.6 V/SCE	at 140 N/cm²	
	CrTiN (1.09-6.79 at.% Ti)				0.535-0.006.5^{A} 0.87-0.074^{K}			4,57 vs. 5.8^{1} a 6.53$^{2, at 150}$	
316L	Cr-N	CFUBMSIP	0.5 M H_2SO_4, 5 ppm HF	70	0.261	-0.13^{D}, -0.29^{S}	2.90^{D}, 0.1^{S}		[99]
	Cr-Al-N (1.86-21.34 at.% Al)				0.277-0.576^{K} 0.057-0.159^{A}	-0.79 to 0.186^{D}, -0.036 to -0.51^{S}	3.55-5.83^{D}, 0.017-0.08^{S}	5.1	
316L	ZrN	Double glow discharge	0.5 M H_2SO_4, 2 ppm HF	70	0.081^{A}, 0.142^{K}	0.847 ^{D}, -1.51^{S}	0.743 ^{D}	7.4 vs. 8.5^{1} a 9.2$^{2, at 150}$	[95]
316L	TaN_x	HPPMS (parameters)	H_2SO_4 (pH=3,pH=6)	80		< 1 (pH 3)^{D}, ~1 (pH 6)^{D}, ~1(pH 3)$^{S, at 1.4 V/SHE}$			[23]
316L	Ta	ICP assisted reactive magnetron sputtering	0.5 M H_2SO_4, 2 ppm HF	80		0.072^{D}, -0.06^{S}	6.15^{D}, 0.2^{S}		[72]
	TaN					0.017-0.12^{D}, -0.06^{S}	0.33-1.06^{D}, 0.2^{S}	11-150 $^{at 150}$	
316L	Ta/TaN	DC reactive magnetron sputtering	0.5 M H_2SO_4, 2 ppm HF	25/80	0.067/0.028	-0.478/-0.182^{D}	1.20/1.53^{D}, 0.21/0.07^{S}	12 vs. 13^{2}	[186]
316L	Cr-C	Unknown	0.5 M H_2SO_4, 5 mg/l HF	80	0.04				[27]
316L	CrC (ratio Cr:C)	CFUBMSIP	0.5 M H_2SO_4, 5 ppm HF	70		10E-5.4^{S} ($Cr_{0.75}C_5$)	1.046-184^{D}, 10E-6.5^{S}	1.4-7.5	[107]
316L	CrC (composition)	PBAIP	0.5 M H_2SO_4, 5 ppm F^-	25/70	0.1-1	0.32-0.1$^{S, at 70 °C}$	0.09/0.23^{D,K} a 3^{D,A}, 0.1-0.03$^{S, at 70 °C}$	2.8 vs. 6.2^{1} a 8.7$^{2, at 120}$	[43]

Material	Surface treatment	Method/technique	Electrolyte	Temperature (°C)	icorr (μA/cm²)			ICR (mΩcm²)	Citation
					at Ecorr	at - 0.1 V/SCE	at 0.6 V/SCE	at 140 N/cm²	
316L	C	CFUBMSIP	0.5 M H_2SO_4, 5 ppm HF	80			1.85^{D}, 2.4^{S}	5.2 vs. 18.4$^{1,2\text{ at }210}$	[9]
316L	CrN	CFUBMSIP	0.5 M H_2SO_4, 2 ppm HF	80			2.14^{D}, 0.25^{S}		[79]
	C/CrN (thickness)						0.5-1.06^{D}, 90-20^{S} pA/cm²	2.6-2.9	
316L	C	Beaming accelerated C60 ions	0.5 M H_2SO_4, 2 ppm HF	80	0.5^{K}, 0.05^{A}	0.5^{S}	0.23^{D}, 0.1^{S}	12 vs. 13^{1,2}	[76]
316L	C on Ti-layer	CFUBMSIP	H_2SO_4 (pH=3), 0.1 ppm HF	80	0.35		0.31$^{S,\text{ at }1.1\text{ V/SHE}}$	14.5/23.4 (po 1.1/1.6 V/SHE)	[22]
	C on Cr-layer				0.76		12.8$^{S,\text{ at }1.1\text{ V/SHE}}$	7.6/34 (po 1.1/1.6 V/SHE)	
	C on Nb-layer				0.54		0.1$^{S,\text{ at }1.1\text{ V/SHE}}$	17.8/26.1 (po 1.1/1.6 V/SHE)	
316L	CrN	CAIP	0.1 M H_2SO_4, 2 ppm HF	80	0.09^{A}, 0.31^{K}	-0.36^{S}	41.4^{D}	23 vs. 25^{1} a 32$^{2,\text{ at }150}$	[187]
	C/CrN				0.12^{A}, 0.07^{K}	-0.18^{S}	1.02^{D}, 0.3^{S}	12 vs. aprox. 12$^{1,2\text{ at }150}$	
316L	C-Cr-N (composition)	CFUBMSIP	0.5 M H_2SO_4, 5 ppm HF	70		1.26-0.16^{S}	0.31-3.72, 0.13-0.016^{S}	2.11-8.23	[121]
316L	CrN/CrNC/C	CFUBMSIP	0.5 M H_2SO_4, 5 ppm HF	70		0.32^{S}	0.61^{D}, 0.025^{S}	2.64	[59]
	CrN						9.63^{D}	15.2	
	CrN/CrNC						4.47^{D}		
316L	C	CFUBMSIP	0.5 M H_2SO_4, 5 ppm HF	70			3.56^{D}	5.4$^{\text{ at }150}$	[123]
	Zr-C/C					-7^{S}	0.49^{D}, 0.06^{S}	3.63 vs. 3.82^{1} a 3.92$^{2,\text{ at }150}$	

Material	Surface treatment	Method/technique	Electrolyte	Temperature (°C)	icorr (μA/cm²) at Ecorr	icorr (μA/cm²) at - 0.1 V/SCE	icorr (μA/cm²) at 0.6 V/SCE	ICR (mΩcm²) at 140 N/cm²	Citation
316L	Poly-p-phenylendiamine	Electrodeposition	0.1 M H_2SO_4		0.78-1.66				[128]
316L	Ag	Unknown	0.5 M H_2SO_4, 2 ppm F^-	80		296^{D}, 30^{S}	593^{D}, 1920^{S}	$5.64^{\text{at 120}}$	[31]
	Ag - passivation					2.27^{D}, 23^{S}	44.4^{D}, 670^{S}	$6.09^{\text{at 120}}$	
316L	Ce enriched		10 g Na_2SO_4 (pH=3.5), 5 ppm F^-	60		-0.15^{S}	0.015^{S}	33	[84]
316	Nb+N	Active screen plasma surface co-alloying	0.5 M H_2SO_4, 2 ppm HF		3.2-20.8		2.21^{S}	8.9-9.4	[73]
316	Pt+N	Active screen plasma co-alloying	0.5 M H_2SO_4, 2 ppm HF	25	24.5-55.7	-26.2 to -14.5^{S}	0.034^{S}	6.3-6.9	[51]
304	TiN	PBAIP	0.5 M H_2SO_4, 2 ppm HF	80	1.45E-2			< 20 vs. $25^{2, \text{at } 240}$	[39]
	Ti_2N/TiN				1.31E-2			< 20 vs. $30^{2, \text{at } 240}$	
304	C	Plasma assisted CVD	0.5 M H_2SO_4, 2 ppm HF	80				14 vs. 16^{1} a 21 vs. $18^{2, \text{at } 150}$	[75]
304	graphen	CVD	3.5% NaCl	25	35.2				[115]
	Ni + graphen	Electroplating + CVD			0.163			30 vs. 36	
304	C	DCMP	0.5 M H_2SO_4, 5 ppm HF				20^{S}		[24]
	Cr/C						$< 0^{S}$	$16.65^{\text{at } 150}$	

Material	Surface treatment	Method/technique	Electrolyte	Temperature (°C)	icorr (μA/cm²)			ICR (mΩcm²)	Citation
					at Ecorr	at - 0.1 V/SCE	at 0.6 V/SCE	at 140 N/cm²	
304	Cr-C (time)	Electroplating	0.5 M H_2SO_4, 2 ppm HF	70	0.27-0.073		1.5E-4[S]	19.52 vs. 26.2[2, at 150]	[53]
304	C-Ni (1 layer)	Plasma blowpipe with internal arc	Unknown		0.45	1.11[D]	3.43[D]	11.81 vs. 8.12	[120]
	C-Ni (2 layers)				1.52E-2	2.37E-2[D]	7.76E-2[D]	5.81 vs. 5.60	
304	PPY	Electrodeposition	0.1 M H_2SO_4 (pH=3),		1				[78]
	PANI				0.1				
304	PPY/PANI	Electrodeposition	0.1 M H_2SO_4, 2 ppm HF	80		206[D]	161[D]		[28]
304	NbN	Plasma surface diffusion alloying	0.05 M H_2SO_4, 2 ppm F^-	70		0.13[D], -0.08 až 0.4[S]	0.071[D], 0.2-0.8[S]	9.26 vs. 18.08[1] a 19.14[2]	[69]
304	NbN	TRD (temp.)	1 M H_2SO_4, 2 ppm HF	80		114-780[D], 2240-3340[S]	70-265[D], 848-1037[S]	30-40	[33]
	Moření + NbN					10.2-12[D], 0.57-4.67[S]	3.2-5.5[D], 0.75-4.5[S]	34-66	
304	MoN	Plasma surface diffusion alloying	0.05 M H_2SO_4, 2 ppm HF	70	4.79[A] 3.83[K]			27.26 vs. 34.25[1] a 43.26[2]	[89]
304	NbC	Plasma surface diffusion alloying	0.5 M H_2SO_4, 2 ppm HF	80	0.06[A] 0.05[K]			8.5 vs 8.8[1] a 9.0[2]	[86]
304	Ni-Mo)	Electrodeposition	H_2SO_4 (pH=5)	70			5.8-12.1[D]	12.77 vs. 92.28[2, at 220]	[37]
	No-Mo-P						30.1-52.9[D]	11.36 vs. 47.32[2, at 220]	

Material	Surface treatment	Method/technique	Electrolyte	Temperature (°C)	icorr (μA/cm²)			ICR (mΩcm²)	Citation
					at Ecorr	at - 0.1 V/SCE	at 0.6 V/SCE	at 140 N/cm²	
Carbon steel	Ni-P	Electroless plating	H_2SO_4 + 0.07 M Na_2SO_4 (pH=4), 2 ppm HF	50		1.45[D]	6.63[D]	16.2	[30]
	CrN	CFUBMSIP				2.99[D]	4.81[D]	2.2	
	Ni-P/CrN	Electroless plating + CFUBMSIP				0.68[D]	0.13[D]	3.3 vs. 3.9[1] a 4.6[2]	
1020	CrN + CrC	Pack chromization	0.5 M H_2SO_4	25	1.24			39.0	[105]
		EDM + pack chromization (2 A/10 A)			0.058/0.576			11.8/17.7	
1045	CrN + CrC	Pack chromization (2 h/4 h)	0.5 M H_2SO_4	25	1.24/0.932			14.9 (pro 2 h) at 150	[104]
		EDM + pack chromization (2 h/4 h)			0.221/0.0286		0.122 (pro 2 h)	9.8 (pro 2 h) at 150	
		Rolling + pack chromization (2 h/ 4 h)			0.0313/0.03	87.5 (pro 2 h)	0.0756[S] (pro 2 h)	5.9 (pro 2 h) at 150	
mild steel	ZnAl (25/75)	Plasma spraying	1 M H_2SO_4	25	6.21				[188]
	ZnAl (50/50)				336				
	ZnAl (75/25)				394				

Explanation to the table: A = anodic conditions (H_2 bubbled), K = cathodic conditions (air/oxygen bubbled), D = potentiodynamic test, S = potentiostatic test, 1 = after potentiostatic test at -0.1 V/SCE, 2 = after potentiostatic test at 0.6 V/SCE

Material	Surface treatment	Method/technique	Electrolyte	Temperature (°C)	icorr (μA/cm²)			ICR (mΩcm²)	Citation
					at Ecorr	at - 0.1 V/SCE	at 0.6 V/SCE	at 140 N/cm²	

Shortcuts for methods: CFUBMSIP = Closed Field Unbalanced Magnetron Sputter Ion Plating, EBPVD = Electron Beam Physical Vapour Deposition, PVD = Physical Vapour Deposition, HPPMS = High Power Pulse Magnetron Sputtering, ICP = Inductively Coupled Plasma, DC = Direct Current, PBAIP = Pulsed Bias Arc Ion Plating, CAIP = Cathode Arc Ion Plating, CVD = Chemical Vapour Deposition, DCMP = Direct Current Magnetron Sputtering, TRD = Thermo-reactive Deposition, EDM = Electrical Discharge Machining

Table S3. Comparison of corrosion resistance and contact resistance of metal materials other than steel.

Material	Surface treatment	Method/technique	Electrolyte	Temp. (°C)	icorr (μA/cm^2)			ICR (mΩcm^2) at 140 N/cm^2	Citat.
					at Ecorr	at - 0.1 V/SCE	at 0.6 V/SCE		
Al 5083	-		0.5 M H_2SO_4, 2 ppm HF	70	1915^{A} 1038^{K}			34 $^{at\ 135}$	[45]
	CrN (3-5 μm)	PVD			$18.85\text{-}57.42^{A}$ $129.2\text{-}79.12^{K}$			6-8.5 $^{at\ 135}$	
Al 5083	-				1915A 1038^{K}				[57]
	CrN	CAE-PVD	0.5 M H_2SO_4, 2 ppm HF	70	$25.02^{A,K}$				
	ZrN/CrN				73.24^{A} 918.9^{K}				
AA 5052	-		0.001 M H_2SO_4, 0.1 ppm NaF, pH=3		268.8		200^{D}	61.58 $^{at\ 150}$	[154]
	TiN	CFUBMSIP			34.4		> 100^{D}	20.08 $^{at\ 150}$	
	CrN				36.8		> 100^{D}	7.76 $^{at\ 150}$	
	C				4.6^{A} 46.7^{K}			6.39 $^{at\ 150}$	
	C/TiN				0.4^{A} 36.0^{K}				
	C/CrN				0.5^{A} 40.7^{K}		aprox 10^{D}	4.08 $^{at\ 150}$	
Al 5052	-		0.5 M H_2SO_4, 2 ppm HF	25	214	110^{D}	5030^{D}		[189]
	Ni-P	Electroless plating			9.37	56.2^{D}	6560^{D}		
	Ni-P-PTFE	Electroless plating			28.1	177^{D}	6010^{D}		
	Ni-P/Au				4.33	0.734^{D}	163^{D}	4	
	Ni-P-PTFE/Au-PTFE				7.58	3.93^{D}	461^{D}	6	
Al 6061	-		0.5 M H_2SO_4, 2 ppm HF		416			184.5	[56]
	CrC	Thermal spraying			65			15.5	
Al	-		0.5 M H_2SO_4, 2 ppm HF	25	74.69			257.25	[158]
Al 6061	-				55.14			128.45	

Material	Surface treatment	Method/technique	Electrolyte	Temp. (°C)	icorr (µA/cm²)			ICR (mΩcm²) at 140 N/cm²	Citat.
					at Ecorr	at - 0.1 V/SCE	at 0.6 V/SCE		
Al 3004	-				44.9			311.25	
Al 1050	-				36.38			361.75	
Al	Ni-P	Electroless plating		25/75	7.59/703.3	5.81E-5/1.97E-3^{S}	1.56E-2/3.59E-3^{S}	83.75	
Al 6061	Ni-P				11.19/3.25E3	9.66E-5/7.13E-3^{S}	1.96E-2/1.18E-2^{S}	84.85	
Al 3004	Ni-P				22.39/3.03E3	1.48E-4/4.47E-3^{S}	1.70E-2/6.52E-3^{S}	56.75	
Al 1050	Ni-P				0.814/576.3	4.37E-6/1.95E-3^{S}	5.95E-5/4.13/E-3^{S}	39.05	
Al 6061	-		0.5 M H_2SO_4	25	53.5				[159]
	Ni-P (1x)	Zincating + electroless plating			10				
	Ni-P (2x)				10				
	Ni-P (3x)				4.4				
Al 5251	-		0.5 M H_2SO_4, 2 ppm HF	25	78		640^{S}		[38]
	Ni-P	Zincating + electroless deposition/electroplating			18.5/1.2	93.4/0.,113^{D}	1.55E4-4.18 ^{D}		
	Ni-Co-P (Ni:Co 5:1/1:1)	Zincating + electroplating			0.732/0.644	1.32/1.20^{D}	31/20.9^{D}		
	Ni-Co-P (Ni:Co 1:1)	Zincating + electroless deposition			14.5/8.96	121/32.1^{D}	7110/640^{D}		
A356	-		0.5 M H_2SO_4, 2 ppm HF			102.94^{D}	842.33^{D}		[19]
	TiN/CrN	DCMS				4.6^{D}, 130^{S}	29.03^{D}, 145^{S}		
Al 7075	-					176.77^{D}	1265.7^{D}		
	TiN/CrN	DCMS				60.29^{D}	89.68^{D}		
GW83	-		1 mM H_2SO_4, 0.1 ppm NaF	70	74.2^{A}, 66.5^{K}			196.5 vs. 354.1$^{2, at 150}$	[58]
	Ni	Electroless deposition			47.8^{A}, 5.6^{K}			126.4 vs. 158.2^{1} a 170.4$^{2, at 150}$	

Material	Surface treatment	Method/technique	Electrolyte	Temp. (°C)	icorr (μA/cm²) at Ecorr	icorr at - 0.1 V/SCE	icorr at 0.6 V/SCE	ICR (mΩcm²) at 140 N/cm²	Citat.
	C	CFUBMSIP			40.1^{A}, 19.9^{K}			23.4 $^{at\ 150}$	
	Ni+C	Electroless deposition + CFUBMSIP			0.8^{A}, 8.65^{K}			2.97 vs. 15.6^{1} a 52.6$^{2,\ at\ 150}$	
Cu	-		0.2M H_2SO_4, 0.1 M HCl, 3 ppm HF	25	51.4				[133]
	PPY	electrodeposition			4.59				
	PPY/PANI				1.62				
Cu-Cr slitina	-		H_2SO_4 (pH=3)	80	42^{A}, 1480^{K}			< 10 $^{at\ 150}$	[172]
	CrN	Thermal nitridation			12.18^{A}, 1480^{K}	1300^{S}	8^{D}	< 10 $^{at\ 150}$	
Inconel 625	-		85% H_3PO_4, 30-120 °C	30-120	0.16-20 μA				[84]
Inconel 825	-				0.13-32 μA				
Hastelloy C-276	-				0.19-24 μA				
Tantalum	-		85% H_3PO_4	120	0.06 μA				
Titanium	-				6300 μA				
Titanium	-		1 mM H_2SO_4, 2 ppm F^-	25	3.39E-3				[185]
Titanium	-		1 M H_2SO_4, 2 ppm F^-		0.063A 0.033K	-2^{S}	1.5^{S}	52.6	[145]
	TiN	DCMS			0.017A 0.019K	-2.5^{S}	1.8^{S}	7.2	
Titanium	-		0.5 M H_2SO_4, 2 ppm HF	25	0.042				[144]
	TiN	Multi-arc ion plating			8.6E-3			2.4 vs. 4.02, at 200	
TiAl6V4	-		0.05 M H_2SO_4 + Na_2SO_4 (pH=1.5 to 3.5), 2 ppm HF	75		719-192^{D}, 740-212^{S}	919-503^{D}, 931-413^{S}	90.6 vs. 101.92, $^{at\ 150}$ (pH1.5)	[29]

Material	Surface treatment	Method/technique	Electrolyte	Temp. (°C)	icorr (μA/cm^2) at Ecorr	at - 0.1 V/SCE	at 0.6 V/SCE	ICR (mΩcm^2) at 140 N/cm^2	Citat.
	Ta$_2$N	Double cathode glow discharge plasma				-0.93 to -0.66^{D}, -0.91 to -0.64^{S}	0.7-0.45^{D}, 0.7-0.35^{S}	10.7 vs. 14.5^{2}, at 150 (pH1.5)	
	-		0.05 M H_2SO_4 + Na_2SO_4 (pH=3.5), 2 ppm HF	25-75		152-192^{D}, 115-212^{S}	185-503^{D}, 245-413^{S}	90.6 vs. 134.5^{2}, at 150 (25 °C)	
	Ta$_2$N	Double cathode glow discharge plasma				-0.35 to -0.66^{D}, -0.36 to -0.64^{S}	0.18-0.45^{D}, 0.16-0.35^{S}	10.7 vs. 20.5^{2}, at 150 (25 °C)	
TiAl6V4	-		0.5 M H_2SO_4, 2-6 ppm HF	70		57.5-219^{D}, 154-569^{S}	851-8190^{D}, 981-6530^{S}	92.9 vs. 121.2^{2}, at 150 (6 ppm)	[64]
	ZrCN	Double cathode glow discharge plasma				-0.33 to -0.56^{D}, -0.34 to -0.55^{S}	15.1-44.7^{D}, 15.7-43.5^{S}	11.2 vs 17.1^{2}, at 150 (6 ppm)	
Ni40Ti40Nb20	-		1 M H_2SO_4	70	0.35^{A} 1.3^{K}	4.7^{D}	2^{D}	54	[166]

Explanation to the table: A = anodic conditions (H_2 bubbled), K = cathodic conditions (air/oxygen bubbled), D = potentiodynamic test, S = potentiostatic test, 1 = after potentiostatic test at -0.1 V/SCE, 2 = after potentiostatic test at 0.6 V/SCE

Shortcut for methods: PVD = Physical Vapour Deposition, CAE-PVD = Cathodic Arc Evaporation Physical Vapour Deposition, CFUBMSIP = Closed Field Unbalanced Magnetron Sputter Ion Plating, DCMS = Direct Current Magnetron Sputtering

References

1. Asri, N.F.; Husaini, T.; Sulong, A.B.; Majlan, E.H.; Daud, W.R.W. Coating of stainless steel and titanium bipolar plates for anticorrosion in PEMFC: A review. *Int. J. Hydrogen Energy* **2017**, *42*, 9135–9148, doi:10.1016/j.ijhydene.2016.06.241.
2. Alo, O.; Otunniyi, I.; Pienaar, H.; Iyuke, S. Materials for Bipolar Plates in Polymer Electrolyte Membrane Fuel Cell: Performance Criteria and Current Benchmarks. *Procedia Manuf.* **2017**, *7*, 395–401, doi:10.1016/j.promfg.2016.12.011.
3. Karimi, S.; Fraser, N.; Roberts, B.; Foulkes, F.R. A Review of Metallic Bipolar Plates for Proton Exchange Membrane Fuel Cells: Materials and Fabrication Methods. *Adv. Mater. Sci. Eng.* **2012**, *2012*, 1–22, doi:10.1155/2012/828070.
4. Taherian, R. RETRACTED: A review of composite and metallic bipolar plates in proton exchange membrane fuel cell: Materials, fabrication, and material selection. *J. Power Sources* **2014**, *265*, 370–390, doi:10.1016/j.jpowsour.2014.04.081.
5. Li, X.; Sabir, I. Review of bipolar plates in PEM fuel cells: Flow-field designs. *Int. J. Hydrogen Energy* **2005**, *30*, 359–371, doi:10.1016/j.ijhydene.2004.09.019.
6. Antunes, R.A.; Oliveira, M.C.L.; Ett, G.; Ett, V. Corrosion of metal bipolar plates for PEM fuel cells: A review. *Int. J. Hydrogen Energy* **2010**, *35*, 3632–3647, doi:10.1016/j.ijhydene.2010.01.059.
7. Daud, W.; Rosli, R.; Majlan, E.; Hamid, S.; Mohamed, R.; Husaini, T. PEM fuel cell system control: A review. *Renew. Energy* **2017**, *113*, 620–638, doi:10.1016/j.renene.2017.06.027.
8. Hermann, A.; Chaudhuri, T.; Spagnol, P. Bipolar plates for PEM fuel cells: A review. *Int. J. Hydrogen Energy* **2005**, *30*, 1297–1302, doi:10.1016/j.ijhydene.2005.04.016.
9. Feng, K.; Cai, X.; Sun, H.; Li, Z.; Chu, P.K. Carbon coated stainless steel bipolar plates in polymer electrolyte membrane fuel cells. *Diam. Relat. Mater.* **2010**, *19*, 1354–1361, doi:10.1016/j.diamond.2010.07.003.
10. Langemann, M.; Fritz, D.L.; Müller, M.; Stolten, D. Validation and characterization of suitable materials for bipolar plates in PEM water electrolysis. *Int. J. Hydrogen Energy* **2015**, *40*, 11385–11391, doi:10.1016/j.ijhydene.2015.04.155.
11. DOE Technical Targets for Polymer Electrolyte Membrane Fuel Cell Components. Available online: https://www.energy.gov/eere/fuelcells/doe-technical-targets-polymer-electrolyte-membrane-fuel-cell-components (accessed on 30 April 2020).
12. Kumar, A.; Ricketts, M.; Hirano, S. Ex situ evaluation of nanometer range gold coating on stainless steel substrate for automotive polymer electrolyte membrane fuel cell bipolar plate. *J. Power Sources* **2010**, *195*, 1401–1407, doi:10.1016/j.jpowsour.2009.09.022.
13. Adrianowycz, O.; Norley, J.; Stuart, D.J.; Flaherty, D.W.; Wayne, R.; Williams, W.; Tietze, R.; Nguyen, Y.-L.H.; A.; Zawodzinski, T.; Pietrasz, P. Next Generation Bipolar Plates for Automotive PEM Fuel Cells. In *Next Generation Bipolar Plates for Automotive PEM Fuel Cells*; Office of Scientific and Technical Information (OSTI), 2010; pp. 849–854.
14. San, F.G.B.; Okur, O. The effect of compression molding parameters on the electrical and physical properties of polymer composite bipolar plates. *Int. J. Hydrogen Energy* **2017**, *42*, 23054–23069, doi:10.1016/j.ijhydene.2017.07.175.
15. Kang, K.; Park, S.; Jo, A.; Lee, K.; Ju, H. Development of ultralight and thin bipolar plates using epoxy-carbon fiber prepregs and graphite composites. *Int. J. Hydrogen Energy* **2017**, *42*, 1691–1697, doi:10.1016/j.ijhydene.2016.05.027.
16. André, J.; Antoni, L.; Petit, J.-P. Corrosion resistance of stainless steel bipolar plates in a PEFC environment: A comprehensive study. *Int. J. Hydrogen Energy* **2010**, *35*, 3684–3697, doi:10.1016/j.ijhydene.2010.01.062.
17. Tawfik, H.; Hung, Y., and Mahajan, D., Metal. bipolar plates for PEM fuel cell - A review. *J. Power Sources* **2007**, *163*, 755–767.
18. Antunes, R.A.; de Oliveira, M.C.; Ett, G.; Ett, V. Carbon materials in composite bipolar plates for polymer electrolyte membrane fuel cells: A review of the main challenges to improve electrical performance. *J. Power Sources* **2011**, *196*, 2945–2961, doi:10.1016/j.jpowsour.2010.12.041.
19. Radzuan, N.A.M.; Sulong, A.B.; Sahari, J. A review of electrical conductivity models for conductive polymer composite. *Int. J. Hydrogen Energy* **2017**, *42*, 9262–9273, doi:10.1016/j.ijhydene.2016.03.045.
20. Kuan, H.-C.; Ma, C.-C.M.; Chen, K.H., and Chen, S.-M., Preparation, electrical, mechanical and thermal properties of composite bipolar plate for a fuel cell. *J. Power Sources* **2004**, *134*, 7–17.
21. Bolouri, A. and Kang, C.G., Study on dimensional and corrosion properties of thixoformed A356 and AA7075 aluminum bipolar plates for proton exchange membrane fuel cells. *Renew. Energy* **2014**, *71*, 616–628.

22. Wang, Y.; Northwood, D.O. Effects of O2 and H2 on the corrosion of SS316L metallic bipolar plate materials in simulated anode and cathode environments of PEM fuel cells. *Electrochimica Acta* **2007**, *52*, 6793–6798, doi:10.1016/j.electacta.2007.05.001.
23. Orsi, A.; Kongstein, O.; Hamilton, P.; Oedegaard, A.; Svenum, I.; Cooke, K. An investigation of the typical corrosion parameters used to test polymer electrolyte fuel cell bipolar plate coatings, with titanium nitride coated stainless steel as a case study. *J. Power Sources* **2015**, *285*, 530–537, doi:10.1016/j.jpowsour.2015.03.111.
24. Bi, F.; Li, X.; Yi, P.; Hou, K.; Peng, L.; Lai, X. Characteristics of amorphous carbon films to resist high potential impact in PEMFCs bipolar plates for automotive application. *Int. J. Hydrogen Energy* **2017**, *42*, 14279–14289, doi:10.1016/j.ijhydene.2017.04.113.
25. Mendizabal, L.; Oedegaard, A.; Kongstein, O.E.; Lædre, S.; Walmsley, J.; Barriga, J.; Gonzalez, J.J. TaNX coatings deposited by HPPMS on SS316L bipolar plates for polymer electrolyte membrane fuel cells: Correlation between corrosion current, contact resistance and barrier oxide film formation. *Int. J. Hydrogen Energy* **2017**, *42*, 3259–3270, doi:10.1016/j.ijhydene.2017.01.070.
26. Mingge, W.; Congda, L.; Tao, H.; Guohai, C.; Donghui, W.; Haifeng, Z.; Dong, Z.; Aiying, W. Chromium interlayer amorphous carbon film for 304 stainless steel bipolar plate of proton exchange membrane fuel cell. *Surf. Coatings Technol.* **2016**, *307*, 374–381, doi:10.1016/j.surfcoat.2016.07.069.
27. Hinds, G.; Brightman, E. Towards more representative test methods for corrosion resistance of PEMFC metallic bipolar plates. *Int. J. Hydrogen Energy* **2015**, *40*, 2785–2791, doi:10.1016/j.ijhydene.2014.12.085.
28. Agneaux, A.; Plouzennec, M.H.; Antoni, L.; Granier, J. Corrosion Behaviour of Stainless Steel Plates in PEMFC Working Conditions. *Fuel Cells* **2006**, *6*, 47–53, doi:10.1002/fuce.200500100.
29. Huang, N.; Yu, H.; Xu, L.; Zhan, S.; Sun, M.; Kirk, D.W. Corrosion kinetics of 316L stainless steel bipolar plate with chromiumcarbide coating in simulated PEMFC cathodic environment. *Results Phys.* **2016**, *6*, 730–736, doi:10.1016/j.rinp.2016.10.002.
30. Ren, Y.J.; Chen, J.; Zeng, C.L.; Li, C., and He, J.J., Electrochemical corrosion characteristics of conducting polypyr-role/polyaniline coatings in simulated environments of a proton exchange membrane fuel cell. *Int. J. Hydrog. Energy* **2016**, *41*, 8542–8549.
31. Ma, J.J.; Xu, J.; Jiang, S.; Munroe, P.; Xie, Z.-H. Effects of pH value and temperature on the corrosion behavior of a Ta2N nanoceramic coating in simulated polymer electrolyte membrane fuel cell environment. *Ceram. Int.* **2016**, *42*, 16833–16851, doi:10.1016/j.ceramint.2016.07.175.
32. Jin, J.; Zheng, D.; Liu, H. The corrosion behavior and mechanical properties of CrN/Ni P multilayer coated mild steel as bipolar plates for proton exchange membrane fuel cells. *Int. J. Hydrogen Energy* **2017**, *42*, 28883–28897, doi:10.1016/j.ijhydene.2017.10.046.
33. Huang, N.; Liang, C.; Wang, H.; Xu, L.; Xu, H. Corrosion Behavior of Passivated 316LSS with Ag Coating as PEMFC Bipolar Plate. *Int. J. Corros.* **2011**, *2011*, 1–7, doi:10.1155/2011/103785.
34. Feng, K.; Wu, G.; Li, Z.; Cai, X.; Chu, P.K. Corrosion behavior of SS316L in simulated and accelerated PEMFC environments. *Int. J. Hydrogen Energy* **2011**, *36*, 13032–13042, doi:10.1016/j.ijhydene.2011.07.058.
35. Dadfar, M.; Salehi, M.; Golozar, M.A., and Trasatti, S., Surface modification of 304 stainless steels to improve corrosion be-havior and interfacial contact resistance of bipolar plates. *Int. J. Hydrog. Energy* **2016**, *41*, 21375–21384.
36. Jin, J.; Zhu, Z.; Zheng, D. Influence of Ti content on the corrosion properties and contact resistance of CrTiN coating in simulated proton exchange membrane fuel cells. *Int. J. Hydrogen Energy* **2017**, *42*, 11758–11770, doi:10.1016/j.ijhydene.2017.02.014.
37. Lim, J.W.; Lee, D.; Kim, M.; Choe, J.; Nam, S.; Gil Lee, D. Composite structures for proton exchange membrane fuel cells (PEMFC) and energy storage systems (ESS): Review. *Compos. Struct.* **2015**, *134*, 927–949, doi:10.1016/j.compstruct.2015.08.121.
38. Yoon, W.; Huang, X.; Fazzino, P.; Reifsnider, K.L.; Akkaoui, M.A. Evaluation of coated metallic bipolar plates for polymer electrolyte membrane fuel cells. *J. Power Sources* **2008**, *179*, 265–273, doi:10.1016/j.jpowsour.2007.12.034.
39. Rajaei, V.; Rashtchi, H.; Raeissi, K.; Shamanian, M. The study of Ni-based nano-crystalline and amorphous alloy coatings on AISI 304 stainless steel for PEM fuel cell bipolar plate application. *Int. J. Hydrogen Energy* **2017**, *42*, 14264–14278, doi:10.1016/j.ijhydene.2017.04.098.
40. Fetohi, A.E.; Hameed, R.A.; El-Khatib, K.; Souaya, E.R. Ni–P and Ni–Co–P coated aluminum alloy 5251 substrates as metallic bipolar plates for PEM fuel cell applications. *Int. J. Hydrogen Energy* **2012**, *37*, 7677–7688, doi:10.1016/j.ijhydene.2012.01.145.
41. Zhang, D.; Duan, L.; Guo, L., and Tuan, W.-H., Corrosion behavior of TiN-coated stainless steel as bipolar plate for proton exchange membrane fuel cell. *Int. J. Hydrog. Energy* **2010**, *35*, 3721–3726.

42. Zhang, D.; Wang, Z.; Huang, K. Composite coatings with in situ formation for Fe–Ni–Cr alloy as bipolar plate of PEMFC. *Int. J. Hydrogen Energy* **2013**, *38*, 11379–11391, doi:10.1016/j.ijhydene.2013.06.112.
43. Richards, J.; Cremers, C.; Fischer, P.; Schmidt, K. Corrosion Studies on Electro Polished Stainless Steels for the Use as Metallic Bipolar Plates in PEMFC Applications. *Energy Procedia* **2012**, *20*, 324–333, doi:10.1016/j.egypro.2012.03.032.
44. Yang, M.; Zhang, D. Effect of surface treatment on the interfacial contact resistance and corrosion resistance of Fe–Ni–Cr alloy as a bipolar plate for polymer electrolyte membrane fuel cells. *Energy* **2014**, *64*, 242–247, doi:10.1016/j.energy.2013.10.080.
45. Wu, B.; Lin, G.; Fu, Y.; Hou, M.; Yi, B. Chromium-containing carbon film on stainless steel as bipolar plates for proton exchange membrane fuel cells. *Int. J. Hydrogen Energy* **2010**, *35*, 13255–13261, doi:10.1016/j.ijhydene.2010.09.036.
46. Fetohi, A.E.; Hameed, R.A.; El–Khatib, K.; Souaya, E.R. Study of different aluminum alloy substrates coated with Ni–Co–P as metallic bipolar plates for PEM fuel cell applications. *Int. J. Hydrogen Energy* **2012**, *37*, 10807–10817, doi:10.1016/j.ijhydene.2012.04.066.
47. Barranco, J.; Barreras, F.; Lozano, A., and Maza, M., Influence of CrN-coating thickness on the corrosion resistance behaviour of aluminium-based bipolar plates. *J. Power Sources* **2011**, *196*, 4283–4289.
48. Sun, H.; Cooke, K.; Eitzinger, G.; Hamilton, P.; Pollet, B. Development of PVD coatings for PEMFC metallic bipolar plates. *Thin Solid Films* **2013**, *528*, 199–204, doi:10.1016/j.tsf.2012.10.094.
49. Gabreab, E.M.; Hinds, G.; Fearn, S.; Hodgson, D.; Millichamp, J.; Shearing, P.R.; Brett, D.J. An electrochemical treatment to improve corrosion and contact resistance of stainless steel bipolar plates used in polymer electrolyte fuel cells. *J. Power Sources* **2014**, *245*, 1014–1026, doi:10.1016/j.jpowsour.2013.07.041.
50. Iversen, A. Stainless steels in bipolar plates—Surface resistive properties of corrosion resistant steel grades during current loads. *Corros. Sci.* **2006**, *48*, 1036–1058, doi:10.1016/j.corsci.2005.05.012.
51. Brady, M.; Elhamid, M.A.; Dadheech, G.; Bradley, J.; Toops, T.; Meyer, H.; Tortorelli, P. Manufacturing and performance assessment of stamped, laser welded, and nitrided FeCrV stainless steel bipolar plates for proton exchange membrane fuel cells. *Int. J. Hydrogen Energy* **2013**, *38*, 4734–4739, doi:10.1016/j.ijhydene.2013.01.143.
52. Kumagai, M.; Myung, S.-T.; Kuwata, S.; Asaishi, R.; Yashiro, H. Corrosion behavior of austenitic stainless steels as a function of pH for use as bipolar plates in polymer electrolyte membrane fuel cells. *Electrochimica Acta* **2008**, *53*, 4205–4212, doi:10.1016/j.electacta.2007.12.078.
53. Lin, K.; Li, X.; Dong, H.; Du, S.; Lu, Y.; Ji, X.; Gu, D. Surface modification of 316 stainless steel with platinum for the application of bipolar plates in high performance proton exchange membrane fuel cells. *Int. J. Hydrogen Energy* **2017**, *42*, 2338–2348, doi:10.1016/j.ijhydene.2016.09.220.
54. Chen, C.-Y.; Su, S.-C. Effects of assembly torque on a proton exchange membrane fuel cell with stamped metallic bipolar plates. *Energy* **2018**, *159*, 440–447, doi:10.1016/j.energy.2018.06.168.
55. Wang, H.-C.; Sheu, H.-H.; Lu, C.-E.; Hou, K.-H.; Ger, M.-D. Preparation of corrosion-resistant and conductive trivalent Cr–C coatings on 304 stainless steel for use as bipolar plates in proton exchange membrane fuel cells by electrodeposition. *J. Power Sources* **2015**, *293*, 475–483, doi:10.1016/j.jpowsour.2015.05.105.
56. Wind, J.; Späh, R.; Kaiser, W.; Böhm, G. Metallic bipolar plates for PEM fuel cells. *J. Power Sources* **2002**, *105*, 256–260, doi:10.1016/s0378-7753(01)00950-8.
57. Brady, M.; Weisbrod, K.; Paulauskas, I.; Buchanan, R.; More, K.; Wang, H.; Wilson, M.; Garzon, F.; Walker, L. Preferential thermal nitridation to form pin-hole free Cr-nitrides to protect proton exchange membrane fuel cell metallic bipolar plates. *Scr. Mater.* **2004**, *50*, 1017–1022, doi:10.1016/j.scriptamat.2003.12.028.
58. Hung, Y.; Tawfik, H.; Mahajan, D. Durability and characterization studies of chromium carbide coated aluminum fuel cell stack. *Int. J. Hydrogen Energy* **2016**, *41*, 12273–12284, doi:10.1016/j.ijhydene.2016.05.136.
59. Barranco, J.; Barreras, F.; Lozano, A.; Lopez, A.M.; Roda, V.; Martín, J.; Maza, M.; Fuentes, G.G.; Almandoz, E.; López-Sabirón, A.M. Cr and Zr/Cr nitride CAE-PVD coated aluminum bipolar plates for polymer electrolyte membrane fuel cells. *Int. J. Hydrogen Energy* **2010**, *35*, 11489–11498, doi:10.1016/j.ijhydene.2010.05.050.
60. Feng, K.; Guo, X.; Li, Z.; Yao, C.; Wu, Y. Investigation of multi-coating process treated magnesium alloy as bipolar plate in polymer electrolyte membrane fuel cell. *Int. J. Hydrogen Energy* **2016**, *41*, 6020–6028, doi:10.1016/j.ijhydene.2016.02.147.
61. Yi, P.; Peng, L.; Zhou, T.; Wu, H.; Lai, X. Cr–N–C multilayer film on 316L stainless steel as bipolar plates for proton exchange membrane fuel cells using closed field unbalanced magnetron sputter ion plating. *Int. J. Hydrogen Energy* **2013**, *38*, 1535–1543, doi:10.1016/j.ijhydene.2012.11.030.
62. Proctor, S.; Linholm, L. A direct measurement of interfacial contact resistance. *IEEE Electron Device Lett.* **1982**, *3*, 294–296, doi:10.1109/edl.1982.25574.

63. Turan, C.; Cora, Ömer, N.; Koç, M. Effect of manufacturing processes on contact resistance characteristics of metallic bipolar plates in PEM fuel cells. *Int. J. Hydrogen Energy* **2011**, *36*, 12370–12380, doi:10.1016/j.ijhydene.2011.06.091.
64. Makkus, R.C.; Janssen, A.H.; A. de Bruijn, F.; Mallant, R.K. Use of stainless steel for cost competitive bipolar plates in the SPFC. *J. Power Sources* **2000**, *86*, 274–282, doi:10.1016/s0378-7753(99)00460-7.
65. Lædre, S.; Kongstein, O.E.; Oedegaard, A.; Seland, F.; Karoliussen, H. Measuring In Situ Interfacial Contact Resistance in a Proton Exchange Membrane Fuel Cell. *J. Electrochem. Soc.* **2019**, *166*, F853–F859, doi:10.1149/2.1511912jes.
66. Xu, J.; Huang, H.J.; Li, Z.; Xu, S.; Tao, H.; Munroe, P.; Xie, Z.-H. Corrosion behavior of a ZrCN coated Ti alloy with potential application as a bipolar plate for proton exchange membrane fuel cell. *J. Alloy. Compd.* **2016**, *663*, 718–730, doi:10.1016/j.jallcom.2015.12.197.
67. Omrani, M.; Habibi, M., and Moti Birjandi, M.S., Enhanced electrical conductivity of two layers AlN-TiN coating on SS316L as bipolar plate using plasma focus device. *Int. J. Hydrog. Energy* **2016**. 41, 5028–5036.
68. Wang, S.; Hou, M.; Zhao, Q.; Jiang, Y.; Wang, Z.; Li, H.; Fu, Y., and Shao, Z., Ti/(Ti,Cr)N/CrN multilayer coated 316L stainless steel by arc ion plating as bipolar plates for proton exchange membrane fuel cells. J. Energy Chem. **2017**. 26, 168–174.
69. Wang, L.; Northwood, D.; Nie, X.; Housden, J.; Spain, E.; Leyland, A.; Matthews, A. Corrosion properties and contact resistance of TiN, TiAlN and CrN coatings in simulated proton exchange membrane fuel cell environments. *J. Power Sources* **2010**, *195*, 3814–3821, doi:10.1016/j.jpowsour.2009.12.127.
70. Liang, P.; Qiu, D.; Peng, L.; Yi, P.; Lai, X.; Ni, J. Contact resistance prediction of proton exchange membrane fuel cell considering fabrication characteristics of metallic bipolar plates. *Energy Convers. Manag.* **2018**, *169*, 334–344, doi:10.1016/j.enconman.2018.05.069.
71. Wang, L.; Sun, J.; Sun, J.; Lv, Y.; Li, S.; Ji, S.; Wen, Z. Niobium nitride modified AISI 304 stainless steel bipolar plate for proton exchange membrane fuel cell. *J. Power Sources* **2012**, *199*, 195–200, doi:10.1016/j.jpowsour.2011.10.034.
72. Dadfar, M.; Salehi, M.; Golozar, M.; Trasatti, S.; Casaletto, M. Surface and corrosion properties of modified passive layer on 304 stainless steel as bipolar plates for PEMFCs. *Int. J. Hydrogen Energy* **2017**, *42*, 25869–25876, doi:10.1016/j.ijhydene.2017.08.169.
73. Shimpalee, S.; Lilavivat, V.; McCrabb, H.; Khunatorn, Y.; Lee, H.-K.; Lee, W.-K.; Weidner, J. Investigation of bipolar plate materials for proton exchange membrane fuel cells. *Int. J. Hydrogen Energy* **2016**, *41*, 13688–13696, doi:10.1016/j.ijhydene.2016.05.163.
74. Choe, C.; Choi, H.; Hong, W.; Lee, J.-J. Tantalum nitride coated AISI 316L as bipolar plate for polymer electrolyte membrane fuel cell. *Int. J. Hydrogen Energy* **2012**, *37*, 405–411, doi:10.1016/j.ijhydene.2011.09.060.
75. Lin, K.; Li, X.; Tian, L.; Dong, H. Active screen plasma surface co-alloying of 316 austenitic stainless steel with both nitrogen and niobium for the application of bipolar plates in proton exchange membrane fuel cells. *Int. J. Hydrogen Energy* **2015**, *40*, 10281–10292, doi:10.1016/j.ijhydene.2015.06.010.
76. Jinlong, L.; Zhuqing, W.; Tongxiang, L.; Ken, S.; Hideo, M. Enhancing the corrosion resistance of the 2205 duplex stainless steel bipolar plates in PEMFCs environment by surface enriched molybdenum. *Results Phys.* **2017**, *7*, 3459–3464, doi:10.1016/j.rinp.2017.09.001.
77. Fukutsuka, T.; Yamaguchi, T.; Miyano, S.-I.; Matsuo, Y.; Sugie, Y.; Ogumi, Z. Carbon-coated stainless steel as PEFC bipolar plate material. *J. Power Sources* **2007**, *174*, 199–205, doi:10.1016/j.jpowsour.2007.08.096.
78. Lee, S.; Pukha, V.; Vinogradov, V.; Kakati, N.; Jee, S.; Cho, S.; Yoon, Y. Nanocomposite-carbon coated at low-temperature: A new coating material for metallic bipolar plates of polymer electrolyte membrane fuel cells. *Int. J. Hydrogen Energy* **2013**, *38*, 14284–14294, doi:10.1016/j.ijhydene.2013.08.013.
79. Wang, Y.; Pu, J.; Wang, J.; Li, J.; Chen, J.; Xue, Q. Interlayer design for the graphite-like carbon film with high load-bearing capacity under sliding-friction condition in water. *Appl. Surf. Sci.* **2014**, *311*, 816–824, doi:10.1016/j.apsusc.2014.05.172.
80. Joseph, S.; McClure, J.; Chianelli, R.; Pich, P.; Sebastian, P. Conducting polymer-coated stainless steel bipolar plates for proton exchange membrane fuel cells (PEMFC). *Int. J. Hydrogen Energy* **2005**, *30*, 1339–1344, doi:10.1016/j.ijhydene.2005.04.011.
81. Feng, K.; Li, Z.; Sun, H.; Yu, L.; Cai, X.; Wu, Y.; Chu, P.K. C/CrN multilayer coating for polymer electrolyte membrane fuel cell metallic bipolar plates. *J. Power Sources* **2013**, *222*, 351–358, doi:10.1016/j.jpowsour.2012.08.087.

82. Mo, J.; Steen, S.M.; Zhang, F.-Y.; Toops, T.J.; Brady, M.P.; Green, J.B. Electrochemical investigation of stainless steel corrosion in a proton exchange membrane electrolyzer cell. *Int. J. Hydrogen Energy* **2015**, *40*, 12506–12511, doi:10.1016/j.ijhydene.2015.07.061.
83. Kumagai, M.; Myung, S.-T.; Katada, Y.; Yashiro, H. Stability of type 310S stainless steel bipolar plates tested at various current densities in proton exchange membrane fuel cells. *Electrochimica Acta* **2016**, *211*, 754–760, doi:10.1016/j.electacta.2016.06.106.
84. Pardo, A.; Merino, M.; Coy, A.E.; Viejo, F.; Arrabal, R.; Matykina, E. Effect of Mo and Mn additions on the corrosion behaviour of AISI 304 and 316 stainless steels in H2SO4. *Corros. Sci.* **2008**, *50*, 780–794, doi:10.1016/j.corsci.2007.11.004.
85. Li, D.; Wang, J.; Chen, D.; Liang, P. Molybdenum addition enhancing the corrosion behaviors of 316 L stainless steel in the simulated cathodic environment of proton exchange membrane fuel cell. *Int. J. Hydrogen Energy* **2015**, *40*, 5947–5957, doi:10.1016/j.ijhydene.2015.01.165.
86. Lavigne, O.; Alémany-Dumont, C.; Normand, B.; Delichère, P.; Descamps, A. Cerium insertion in 316L passive film: Effect on conductivity and corrosion resistance performances of metallic bipolar plates for PEM fuel cell application. *Surf. Coatings Technol.* **2010**, *205*, 1870–1877, doi:10.1016/j.surfcoat.2010.08.051.
87. Yun, Y.-H. Deposition of gold–titanium and gold–nickel coatings on electropolished 316L stainless steel bipolar plates for proton exchange membrane fuel cells. *Int. J. Hydrogen Energy* **2010**, *35*, 1713–1718, doi:10.1016/j.ijhydene.2009.12.036.
88. Wang, L.; Sun, J.; Kang, B.; Li, S.; Ji, S.; Wen, Z.; Wang, X. Electrochemical behaviour and surface conductivity of niobium carbide-modified austenitic stainless steel bipolar plate. *J. Power Sources* **2014**, *246*, 775–782, doi:10.1016/j.jpowsour.2013.08.025.
89. Turan, C.; Cora, Ömer, N.; Koç, M. Contact resistance characteristics of coated metallic bipolar plates for PEM fuel cells – investigations on the effect of manufacturing. *Int. J. Hydrogen Energy* **2012**, *37*, 18187–18204, doi:10.1016/j.ijhydene.2012.09.042.
90. Liu, C.; Bi, Q.; Leyland, A.; Matthews, A. An electrochemical impedance spectroscopy study of the corrosion behaviour of PVD coated steels in 0.5 N NaCl aqueous solution: Part II. *Corros. Sci.* **2003**, *45*, 1257–1273, doi:10.1016/s0010-938x(02)00214-7.
91. Wang, L.; Sun, J.; Li, P.; Sun, J.; Lv, Y.; Jing, B.; Li, S.; Ji, S.; Wen, Z. Molybdenum nitride modified AISI 304 stainless steel bipolar plate for proton exchange membrane fuel cell. *Int. J. Hydrogen Energy* **2012**, *37*, 5876–5883, doi:10.1016/j.ijhydene.2011.12.147.
92. Lee, W.-J.; Yun, E.-Y.; Lee, H.-B.-R.; Hong, S.W., and Kwon, S.-H., Ultrathin effective TiN protective films prepared by plas-ma-enhanced atomic layer deposition for high performance metallic bipolar plates of polymer electrolyte membrane fuel cells. *Appl. Surf. Sci.* **2020**, *519*, 146215.
93. Fu, Y.; Lin, G.; Hou, M.; Wu, B.; Li, H.; Hao, L.; Shao, Z.; Yi, B. Optimized Cr-nitride film on 316L stainless steel as proton exchange membrane fuel cell bipolar plate. *Int. J. Hydrogen Energy* **2009**, *34*, 453–458, doi:10.1016/j.ijhydene.2008.09.104.
94. Shen, H.; Wang, L., and Sun, J., Characteristics and properties of CrN compound layer produced by plasma nitriding of Cr-electroplated of AISI 304 stainless steel. *Surf. Coat. Tech.* **2020**, *385*, 125450.
95. Haye, E.; Deschamps, F.; Caldarella, G.; Piedboeuf, M.-L.; Lafort, A.; Cornil, H.; Colomer, J.-F.; Pireaux, J.-J.; Job, N. Formable chromium nitride coatings for proton exchange membrane fuel cell stainless steel bipolar plates. *Int. J. Hydrogen Energy* **2020**, *45*, 15358–15365, doi:10.1016/j.ijhydene.2020.03.248.
96. Yang, L.X.; Liu, R.J.; Wang, Y.; Liu, H.J.; Zeng, C.L.; Fu, C. Growth of nanocrystalline β-Nb2N coating on 430 ferritic stainless steel bipolar plates of PEMFCs by disproportionation reaction of Nb(IV) ions in molten salt. *Corros. Sci.* **2020**, *174*, 108862, doi:10.1016/j.corsci.2020.108862.
97. Xu, J.; Xu, S.; Munroe, P.; Xie, Z.-H. A ZrN nanocrystalline coating for polymer electrolyte membrane fuel cell metallic bipolar plates prepared by reactive sputter deposition. *RSC Adv.* **2015**, *5*, 67348–67356, doi:10.1039/c5ra09733a.
98. Song, M.; Guo, J.; Yang, Y.; Geng, K.; Xiang, M.; Zhu, Q.; Hu, C.; Zhao, H. Fe2Ti interlayer for improved adhesion strength and corrosion resistance of TiN coating on stainless steel 316L. *Appl. Surf. Sci.* **2020**, *504*, 144483, doi:10.1016/j.apsusc.2019.144483.
99. Zhang, M.; Kim, K.H.; Shao, Z.; Wang, F.; Zhao, S.; Suo, N. Effects of Mo content on microstructure and corrosion resistance of arc ion plated Ti–Mo–N films on 316L stainless steel as bipolar plates for polymer exchange membrane fuel cells. *J. Power Sources* **2014**, *253*, 201–204, doi:10.1016/j.jpowsour.2013.12.075.
100. Jin, J.; Hu, M.; Zhao, X. Investigation of incorporating oxygen into TiN coating to resist high potential effects on PEMFC bipolar plates in vehicle applications. *Int. J. Hydrogen Energy* **2020**, *45*, 23310–23326, doi:10.1016/j.ijhydene.2020.06.059.

101. Bi, F.; Yi, P.; Zhou, T.; Peng, L.; Lai, X. Effects of Al incorporation on the interfacial conductivity and corrosion resistance of CrN film on SS316L as bipolar plates for proton exchange membrane fuel cells. *Int. J. Hydrogen Energy* **2015**, *40*, 9790–9802, doi:10.1016/j.ijhydene.2015.06.012.
102. Ingle, A.V.; Raja, V.S.; Rangarajan, J., and Mishra, P., Corrosion resistant quaternary Al–Cr–Mo–N coating on type 316L stainless steel bipolar plates for proton exchange membrane fuel cells. *Int. J. Hydrog. Energy* **2020**, *45*, 3094–3107.
103. Dong, Z.; Zhou, T.; Liu, J.; Zhang, X.; Shen, B.; Hu, W.; Liu, L. Performance of surface chromizing layer on 316L stainless steel for proton exchange membrane fuel cell bipolar plates. *Int. J. Hydrogen Energy* **2019**, *44*, 22110–22121, doi:10.1016/j.ijhydene.2019.06.099.
104. Dong, Z.; Zhou, T.; Liu, J.; Zhang, X.; Shen, B.; Hu, W.; Liu, L. Effects of pack chromizing on the microstructure and anticorrosion properties of 316L stainless steel. *Surf. Coatings Technol.* **2019**, *366*, 86–96, doi:10.1016/j.surfcoat.2019.03.022.
105. Yang, L.; Yu, H.; Jiang, L.; Zhu, L.; Jian, X.; Wang, Z. Improved anticorrosion properties and electrical conductivity of 316L stainless steel as bipolar plate for proton exchange membrane fuel cell by lower temperature chromizing treatment. *J. Power Sources* **2010**, *195*, 2810–2814, doi:10.1016/j.jpowsour.2009.11.018.
106. Bai, C.-Y.; Wen, T.-M.; Hou, K.-H.; Ger, M.-D. The bipolar plate of AISI 1045 steel with chromized coatings prepared by low-temperature pack cementation for proton exchange membrane fuel cell. *J. Power Sources* **2010**, *195*, 779–786, doi:10.1016/j.jpowsour.2009.08.036.
107. Bai, C.-Y.; Ger, M.-D.; Wu, M.-S. Corrosion behaviors and contact resistances of the low-carbon steel bipolar plate with a chromized coating containing carbides and nitrides. *Int. J. Hydrogen Energy* **2009**, *34*, 6778–6789, doi:10.1016/j.ijhydene.2009.05.103.
108. Wang, H.-C.; Hou, K.-H.; Lu, C.-E.; Ger, M.-D. The study of electroplating trivalent CrC alloy coatings with different current densities on stainless steel 304 as bipolar plate of proton exchange membrane fuel cells. *Thin Solid Films* **2014**, *570*, 209–214, doi:10.1016/j.tsf.2014.03.034.
109. Zhao, Y.; Wei, L.; Yi, P.; Peng, L. Influence of Cr-C film composition on electrical and corrosion properties of 316L stainless steel as bipolar plates for PEMFCs. *Int. J. Hydrogen Energy* **2016**, *41*, 1142–1150, doi:10.1016/j.ijhydene.2015.10.047.
110. Wang, L.; Tao, Y.; Zhang, Z.; Wang, Y.; Feng, Q.; Wang, H.; Li, H. Molybdenum carbide coated 316L stainless steel for bipolar plates of proton exchange membrane fuel cells. *Int. J. Hydrogen Energy* **2019**, *44*, 4940–4950, doi:10.1016/j.ijhydene.2018.12.184.
111. Abbas, N.; Qin, X.; Ali, S.; Zhu, G.; Lu, J.; E.; Alam, F.; Wattoo, A.G.; Zeng, X.; Gu, K.; Tang, J. Direct deposition of extremely low interface-contact-resistant Ti2AlC MAX-phase coating on stainless-steel by mid-frequency magnetron sputtering method. *J. Eur. Ceram. Soc.* **2020**, *40*, 3338–3342, doi:10.1016/j.jeurceramsoc.2020.02.033.
112. Abbas, N.; Qin, X.; Ali, S.; Zhu, G.; Yi, Z.; Yang, X.; Zeng, X.; Ullah, Z.; Gu, K.; Tang, J. Study of microstructural variation with annealing temperature of Ti–Al–C films coated on stainless steel substrates. *Int. J. Hydrogen Energy* **2020**, *45*, 3186–3192, doi:10.1016/j.ijhydene.2019.11.163.
113. Lu, J.; Abbas, N.; Tang, J.; Zhu, G. Synthesis and characterization of conductive ceramic MAX-phase coatings for metal bipolar plates in simulated PEMFC environments. *Corros. Sci.* **2019**, *158*, doi:10.1016/j.corsci.2019.108106.
114. Lu, J.; Abbas, N.; Tang, J.; Hu, R.; Zhu, G. Characterization of Ti3SiC2-coating on stainless steel bipolar plates in simulated proton exchange membrane fuel cell environments. *Electrochem. Commun.* **2019**, *105*, 106490, doi:10.1016/j.elecom.2019.106490.
115. Li, H.; Guo, P.; Zhang, D.; Liu, L.; Wang, Z.; Ma, G.; Xin, Y.; Ke, P.; Saito, H.; Wang, A. Interface-induced degradation of amorphous carbon films/stainless steel bipolar plates in proton exchange membrane fuel cells. *J. Power Sources* **2020**, *469*, 228269, doi:10.1016/j.jpowsour.2020.228269.
116. Chung, C.-Y.; Chen, S.-K.; Chiu, P.-J.; Chang, M.-H.; Hung, T.-T.; Ko, T.-H. Carbon film-coated 304 stainless steel as PEMFC bipolar plate. *J. Power Sources* **2008**, *176*, 276–281, doi:10.1016/j.jpowsour.2007.10.022.
117. Pu, N.-W.; Shi, G.-N.; Liu, Y.-M.; Sun, X.; Chang, J.-K.; Sun, C.-L.; Ger, M.-D.; Chen, C.-Y.; Wang, P.-C.; Peng, Y.-Y.; et al. Graphene grown on stainless steel as a high-performance and ecofriendly anti-corrosion coating for polymer electrolyte membrane fuel cell bipolar plates. *J. Power Sources* **2015**, *282*, 248–256, doi:10.1016/j.jpowsour.2015.02.055.
118. Hou, K.; Yi, P.; Peng, L.; Lai, X. Niobium doped amorphous carbon film on metallic bipolar plates for PEMFCs: First principle calculation, microstructure and performance. *Int. J. Hydrogen Energy* **2019**, *44*, 3144–3156, doi:10.1016/j.ijhydene.2018.12.040.
119. Zhang, D.; Yi, P.; Peng, L.; Lai, X.; Pu, J. Amorphous carbon films doped with silver and chromium to achieve ultra-low interfacial electrical resistance and long-term durability in the application of proton exchange membrane fuel cells. *Carbon* **2019**, *145*, 333–344, doi:10.1016/j.carbon.2019.01.050.

120. Alaefour, I.; Shahgaldi, S.; Zhao, J.; Li, X. Synthesis and Ex-Situ characterizations of diamond-like carbon coatings for metallic bipolar plates in PEM fuel cells. *Int. J. Hydrogen Energy* **2021**, *46*, 11059–11070, doi:10.1016/j.ijhydene.2020.09.259.
121. Che, J.; Yi, P.; Peng, L.; Lai, X. Impact of pressure on carbon films by PECVD toward high deposition rates and high stability as metallic bipolar plate for PEMFCs. *Int. J. Hydrogen Energy* **2020**, *45*, 16277–16286, doi:10.1016/j.ijhydene.2020.04.078.
122. Wlodarczyk, R.; Zasada, D.; Morel, S.; Kacprzak, A. A comparison of nickel coated and uncoated sintered stainless steel used as bipolar plates in low-temperature fuel cells. *Int. J. Hydrogen Energy* **2016**, *41*, 17644–17651, doi:10.1016/j.ijhydene.2016.07.231.
123. Yi, P.; Peng, L.; Zhou, T.; Huang, J.; Lai, X. Composition optimization of multilayered chromium-nitride–carbon film on 316L stainless steel as bipolar plates for proton exchange membrane fuel cells. *J. Power Sources* **2013**, *236*, 47–53, doi:10.1016/j.jpowsour.2013.02.034.
124. Fu, Y.; Lin, G.; Hou, M.; Wu, B.; Shao, Z.; Yi, B. Carbon-based films coated 316L stainless steel as bipolar plate for proton exchange membrane fuel cells. *Int. J. Hydrogen Energy* **2009**, *34*, 405–409, doi:10.1016/j.ijhydene.2008.10.068.
125. Bi, F.; Peng, L.; Yi, P.; Lai, X. Multilayered Zr–C/a-C film on stainless steel 316L as bipolar plates for proton exchange membrane fuel cells. *J. Power Sources* **2016**, *314*, 58–65, doi:10.1016/j.jpowsour.2016.02.078.
126. Li, X.; Hou, K.; Qiu, D.; Yi, P.; Lai, X. A first principles and experimental study on the influence of nitrogen doping on the performance of amorphous carbon films for proton exchange membrane fuel cells. *Carbon* **2020**, *167*, 219–229, doi:10.1016/j.carbon.2020.05.082.
127. Dong, H.; He, S.; Wang, X.; Zhang, C.; Sun, D. Study on conductivity and corrosion resistance of N-doped and Cr/N co-doped DLC films on bipolar plates for PEMFC. *Diam. Relat. Mater.* **2020**, *110*, 108156, doi:10.1016/j.diamond.2020.108156.
128. Jiang, L.; Syed, J.A.; Gao, Y.; Zhang, Q.; Zhao, J.; Lu, H.; Meng, X. Electropolymerization of camphorsulfonic acid doped conductive polypyrrole anti-corrosive coating for 304SS bipolar plates. *Appl. Surf. Sci.* **2017**, *426*, 87–98, doi:10.1016/j.apsusc.2017.07.077.
129. García, M.L.; Smit, M.A. Study of electrodeposited polypyrrole coatings for the corrosion protection of stainless steel bipolar plates for the PEM fuel cell. *J. Power Sources* **2006**, *158*, 397–402, doi:10.1016/j.jpowsour.2005.09.037.
130. Shanmugham, C.; Rajendran, N. Corrosion resistance of poly p-phenylenediamine conducting polymer coated 316L SS bipolar plates for Proton Exchange Membrane Fuel Cells. *Prog. Org. Coatings* **2015**, *89*, 42–49, doi:10.1016/j.porgcoat.2015.07.023.
131. Jiang, L.; Syed, J.A.; Lu, H.; Meng, X. In-situ electrodeposition of conductive polypyrrole-graphene oxide composite coating for corrosion protection of 304SS bipolar plates. *J. Alloy. Compd.* **2019**, *770*, 35–47, doi:10.1016/j.jallcom.2018.07.277.
132. Jiang, L.; Syed, J.A.; Zhang, G.; Ma, Y.; Ma, J.; Lu, H.; Meng, X. Enhanced anticorrosion performance of PPY-graphene oxide/PPY-camphorsulfonic acid composite coating for 304SS bipolar plates in proton exchange membrane fuel cell. *J. Ind. Eng. Chem.* **2019**, *80*, 497–507, doi:10.1016/j.jiec.2019.08.032.
133. Sharma, S.; Zhang, K.; Gupta, G., and Santamaria, D.G., Exploring PANI-TiN nanoparticle coatings in a PEFC environment: Enhancing corrosion resistance and conductivity of stainless steel bipolar plates. *Energies* **2017**, *10*, 1152–1152.
134. Wang, Y.; Zhang, S.; Wang, P.; Lu, Z.; Chen, S., and Wang, L., Synthesis and corrosion protection of Nb doped TiO2 na-nopowders modified polyaniline coating on 316 stainless steel bipolar plates for proton-exchange membrane fuel cells. *Prog. Org. Coat.* **2019**, 137, 105327.
135. Pan, T.; Zuo, X.; Wang, T.; Hu, J.; Chen, Z.; Ren, Y. Electrodeposited conductive polypyrrole/polyaniline composite film for the corrosion protection of copper bipolar plates in proton exchange membrane fuel cells. *J. Power Sources* **2016**, *302*, 180–188, doi:10.1016/j.jpowsour.2015.10.027.
136. Akula, S.; Kalaiselvi, P.; Sahu, A.K.; Chellammal, S. Electrodeposition of conductive PAMT/PPY bilayer composite coatings on 316L stainless steel plate for PEMFC application. *Int. J. Hydrogen Energy* **2021**, *46*, 17909–17921, doi:10.1016/j.ijhydene.2021.02.196.
137. Pan, T.; Chen, Y.; Zhang, B.; Hu, J.; Li, C. Corrosion behavior of niobium coated 304 stainless steel in acid solution. *Appl. Surf. Sci.* **2016**, *369*, 320–325, doi:10.1016/j.apsusc.2016.02.088.
138. Mohammadi, N.; Yari, M., and Allahkaram, S.R., Characterization of PbO2 coating electrodeposited onto stainless steel 316L substrate for using as PEMFC's bipolar plates. *Surf. Coat. Tech.* **2013**, *236*, 341–346.
139. Wang, H.; Turner, J.A.; Li, X.; Teeter, G. Process modification for coating SnO2:F on stainless steels for PEM fuel cell bipolar plates. *J. Power Sources* **2008**, *178*, 238–247, doi:10.1016/j.jpowsour.2007.12.010.

140. Manso, A.; Marzo, F.; Garicano, X.; Alegre, C.; Lozano, A.; Barreras, F. Corrosion behavior of tantalum coatings on AISI 316L stainless steel substrate for bipolar plates of PEM fuel cells. *Int. J. Hydrogen Energy* **2020**, *45*, 20679–20691, doi:10.1016/j.ijhydene.2019.12.157.
141. Wang, X.-Z.; Fan, H.-Q.; Muneshwar, T.; Cadien, K.; Luo, J.-L. Balancing the corrosion resistance and through-plane electrical conductivity of Cr coating via oxygen plasma treatment. *J. Mater. Sci. Technol.* **2021**, *61*, 75–84, doi:10.1016/j.jmst.2020.06.012.
142. Chanda, U.K.; Padhee, S.P.; Pandey, A.K.; Roy, S., and Pati, S., Electrodeposited Ni–Mo.–Cr–P coatings for AISI 1020 steel bipolar plates. *Int. J. Hydrog. Energy* **2020**, *45*, 21892–21904.
143. He, R.; Jiang, J.; Wang, R.; Yue, Y.; Chen, Y.; Pan, T. Anti-corrosion and conductivity of titanium diboride coating on metallic bipolar plates. *Corros. Sci.* **2020**, *170*, 108646, doi:10.1016/j.corsci.2020.108646.
144. Jin, C.K.; Jeong, M.G., and Kang, C.G., Fabrication of titanium bipolar plates by rubber forming and performance of single cell using TiN-coated titanium bipolar plates. *Int. J. Hydrog. Energy* 2014. *39*, 21480–21488.
145. El-Enin, S.A.A.; Abdel-Salam, O.E.; El-Abd, H.; Amin, A.M. New electroplated aluminum bipolar plate for PEM fuel cell. *J. Power Sources* **2008**, *177*, 131–136, doi:10.1016/j.jpowsour.2007.11.042.
146. Zhang, D.; Duan, L.; Guo, L.; Wang, Z.; Zhao, J.; Tuan, W.-H., and Niihara, K., TiN-coated titanium as the bipolar plate for PEMFC by multi-arc ion plating. *Int. J. Hydrog. Energy*, **2011**, *36*, 9155–9161.
147. Lee, K.H.; Jin, C.K.; Kang, C.G.; Seo, H.Y.; Kim, J.D. Fabrication of Titanium Bipolar Plates by Rubber Forming Process and Evaluation Characteristics of TiN coated Titanium Bipolar Plates. *Fuel Cells* **2014**, *15*, 170–177, doi:10.1002/fuce.201400091.
148. Bi, J.; Yang, J.; Liu, X.; Wang, D.; Yang, Z.; Liu, G.; Wang, X. Development and evaluation of nitride coated titanium bipolar plates for PEM fuel cells. *Int. J. Hydrogen Energy* **2021**, *46*, 1144–1154, doi:10.1016/j.ijhydene.2020.09.217.
149. Zhang, P.; Hao, C.; Han, Y.; Du, F.; Wang, H.; Wang, X.; Sun, J. Electrochemical behavior and surface conductivity of NbC modified Ti bipolar plate for proton exchange membrane fuel cell. *Surf. Coatings Technol.* **2020**, *397*, 126064, doi:10.1016/j.surfcoat.2020.126064.
150. Shi, J.; Zhang, P.; Han, Y.; Wang, H.; Wang, X.; Yu, Y.; Sun, J. Investigation on electrochemical behavior and surface conductivity of titanium carbide modified Ti bipolar plate of PEMFC. *Int. J. Hydrogen Energy* **2020**, *45*, 10050–10058, doi:10.1016/j.ijhydene.2020.01.203.
151. Jin, J.; He, Z.; Zhao, X. Formation of a protective TiN layer by liquid phase plasma electrolytic nitridation on Ti–6Al–4V bipolar plates for PEMFC. *Int. J. Hydrogen Energy* **2020**, *45*, 12489–12500, doi:10.1016/j.ijhydene.2020.02.152.
152. Peng, S.; Xu, J.; Li, Z.; Jiang, S.; Munroe, P.; Xie, Z.-H.; Lu, H. A reactive-sputter-deposited TiSiN nanocomposite coating for the protection of metallic bipolar plates in proton exchange membrane fuel cells. *Ceram. Int.* **2020**, *46*, 2743–2757, doi:10.1016/j.ceramint.2019.09.263.
153. Joseph, S.; McClure, J.; Sebastian, P.; Moreira, J.; Valenzuela, E. Polyaniline and polypyrrole coatings on aluminum for PEM fuel cell bipolar plates. *J. Power Sources* **2008**, *177*, 161–166, doi:10.1016/j.jpowsour.2007.09.113.
154. Lee, C.-H.; Lee, Y.-B.; Kim, K.-M.; Jeong, M.-G., and Lim, D.-S., Electrically conductive polymer composite coating on aluminum for PEM fuel cells bipolar plate. *Renew. Energy* **2013**, *54*, 46–50.
155. Mawdsley, J.R.; Carter, J.D.; Wang, X.; Niyogi, S.; Fan, C.Q.; Koc, R.; Osterhout, G. Composite-coated aluminum bipolar plates for PEM fuel cells. *J. Power Sources* **2013**, *231*, 106–112, doi:10.1016/j.jpowsour.2012.12.074.
156. Li, Z.; Feng, K.; Wang, Z.; Cai, X.; Yao, C.; Wu, Y. Investigation of single-layer and multilayer coatings for aluminum bipolar plate in polymer electrolyte membrane fuel cell. *Int. J. Hydrogen Energy* **2014**, *39*, 8421–8430, doi:10.1016/j.ijhydene.2014.03.136.
157. Silva, F.C.; Prada Ramirez, O.M.; Tunes, M.A.; Edmondson, P.D.; Sagás, J.C.; Fontana, L.C.; de Melo, H.G., and Schön, C.G., Corrosion resistance of functionally graded TiN/Ti coatings for proton exchange membrane fuel cells. *Int. J. Hydrog. Energy* **2020**, *45*, 33993–34010.
158. 158. Chiang, T.-Y.; Ay, S.; Tsai, L.-C.; Sheu, H.-H., and Lu, C.-E., Corrosion resistance of 5052 Al-alloy with a zirconia-rich conversion coating used in bipolar plates in PEMFCs. Int. J. Electrochem. Sci., 2014. 9(11): P. 5850-5863, 14 pp.
159. Hua, Q.; Zeng, Y.; He, Z.; Xu, Q.; Min, Y. Microstructure, synergistic mechanism and corrosion behavior of tin oxide conversion film modified by chitosan on aluminum alloy surface. *Colloid Interface Sci. Commun.* **2020**, *36*, 100262, doi:10.1016/j.colcom.2020.100262.

160. Fetohi, A.E.; Hameed, R.A.; El-Khatib, K. Development of electroless Ni–P modified aluminum substrates in a simulated fuel cell environment. *J. Ind. Eng. Chem.* **2015**, *30*, 239–248, doi:10.1016/j.jiec.2015.05.028.
161. Gutiérrez, A.G.G.; Pech-Canul, M.A.; Sebastian, P.J. Zincating Effect on Corrosion Resistance of Electroless Ni-P Coating on Aluminum Alloy 6061. *Fuel Cells* **2017**, *17*, 770–777, doi:10.1002/fuce.201600212.
162. Marzo, F.; Alberro, M.; Manso, A.; Garikano, X.; Alegre, C.; Montiel, M.; Lozano, A.; Barreras, F. Evaluation of the corrosion resistance of Ni(P)Cr coatings for bipolar plates by electrochemical impedance spectroscopy. *Int. J. Hydrogen Energy* **2020**, *45*, 20632–20646, doi:10.1016/j.ijhydene.2020.03.076.
163. Nikiforov, A.; Petrushina, I.; Christensen, E.; Tomás-García, A.; Bjerrum, N. Corrosion behaviour of construction materials for high temperature steam electrolysers. *Int. J. Hydrogen Energy* **2011**, *36*, 111–119, doi:10.1016/j.ijhydene.2010.09.023.
164. Ghorbani, M.M.; Taherian, R., and Bozorg, M., Investigation on physical and electrochemical properties of TiN-coated Monel alloy used for bipolar plates of proton exchange membrane fuel cell. *Mater. Chem. Phys.* **2019**, *238*, 121916.
165. Ben Jadi, S.; El Jaouhari, A.; Aouzal, Z.; El Guerraf, A.; Bouabdallaoui, M.; Wang, R.; Bazzaoui, E.A., and Bazzaoui, M., Elec-tropolymerization and corrosion resistance of polypyrrole on nickel bipolar plate for PEM fuel cell application. Mater. Today **2020**, *22*, 52–56.
166. Park, J.; Kusumah, P.; Kim, Y.; Kim, K.; Kwon, K., and Lee, C.K., Corrosion Prevention of Chromium Nitride Coating with an Application to Bipolar Plate Materials. *Electrochemistry* **2014**, 658–662.
167. Park, J.; Dilasari, B.; Kim, Y.; Kim, K.; Lee, C.K.; Kwon, K. Passivation Behavior and Surface Resistance of Electrodeposited Nickel-Carbon Composites. *Electrochem.* **2014**, *82*, 561–565, doi:10.5796/electrochemistry.82.561.
168. Thalmaier, G.; Vida-Simiti, I.; Vermesan, H.; Codrean, C.; Chira, M. Corrosion Resistance Measurements of Amorphous Ni40Ti40Nb20 Bipolar Plate Material for Polymer Electrolyte Membrane Fuel Cells. *Adv. Eng. Forum* **2013**, *8-9*, 335–342, doi:10.4028/www.scientific.net/aef.8-9.335.
169. Yokoyama, M.; Yamaura, S.-I.; Kimura, H.; Inoue, A. Production of metallic glassy bipolar plates for PEM fuel cells by hot pressing in the supercooled liquid state. *Int. J. Hydrogen Energy* **2008**, *33*, 5678–5685, doi:10.1016/j.ijhydene.2008.07.034.
170. Tian, R.; Qin, Z. Bulk metallic glass Zr55Cu30Al10Ni5 bipolar plates for proton exchange membrane fuel cell. *Energy Convers. Manag.* **2014**, *86*, 927–932, doi:10.1016/j.enconman.2014.06.035.
171. Lv, J.; Tongxiang, L.; Chen, W. The effects of molybdenum and reduced graphene oxide on corrosion resistance of amorphous nickel–phosphorus as bipolar plates in PEMFC environment. *Int. J. Hydrogen Energy* **2016**, *41*, 9738–9745, doi:10.1016/j.ijhydene.2016.03.104.
172. Han, X.; Feng, S.; Chen, S.; Cao, Q.; Zhang, C.; Chen, Q. An investigation of Zr-based bulk metallic glasses as bipolar plates for proton exchange membrane fuel cells. *Int. J. Hydrogen Energy* **2020**, *45*, 3132–3144, doi:10.1016/j.ijhydene.2019.11.174.
173. Nikam, V.V.; Reddy, R.G. Corrosion studies of a copper–beryllium alloy in a simulated polymer electrolyte membrane fuel cell environment. *J. Power Sources* **2005**, *152*, 146–155, doi:10.1016/j.jpowsour.2005.02.035.
174. Lee, H.-Y.; Lee, S.-H.; Kim, J.-H.; Kim, M.-C.; Wee, D.-M. Thermally nitrided Cu–5.3Cr alloy for application as metallic separators in PEMFCs. *Int. J. Hydrogen Energy* **2008**, *33*, 4171–4177, doi:10.1016/j.ijhydene.2008.04.057.
175. Lee, Y.H.; Noh, S.; Lee, J.-H.; Chun, S.-H.; Cha, S.W.; Chang, I. Durable graphene-coated bipolar plates for polymer electrolyte fuel cells. *Int. J. Hydrogen Energy* **2017**, *42*, 27350–27353, doi:10.1016/j.ijhydene.2017.09.053.
176. Sadeghian, Z.; Hadidi, M.R.; Salehzadeh, D., and Nemati, A., Hydrophobic octadecylamine-functionalized graphene/TiO2 hybrid coating for corrosion protection of copper bipolar plates in simulated proton exchange membrane fuel cell envi-ronment. *Int. J. Hydrog. Energy* **2020**, 45, 15380–15389.
177. Altun, H.; Sen, S. The effect of PVD coatings on the corrosion behaviour of AZ91 magnesium alloy. *Mater. Des.* **2006**, *27*, 1174–1179, doi:10.1016/j.matdes.2005.02.004.
178. Wu, G.; Zeng, X.; Ding, W.; Guo, X.; Yao, S. Characterization of ceramic PVD thin films on AZ31 magnesium alloys. *Appl. Surf. Sci.* **2006**, *252*, 7422–7429, doi:10.1016/j.apsusc.2005.08.095.
179. Hollstein, F.; Wiedemann, R.; Scholz, J. Characteristics of PVD-coatings on AZ31hp magnesium alloys. *Surf. Coatings Technol.* **2003**, *162*, 261–268, doi:10.1016/s0257-8972(02)00671-0.
180. Mao, Y.; Li, Z.; Feng, K.; Guo, X.; Zhou, Z.; Dong, J.; Wu, Y. Preparation, characterization and wear behavior of carbon coated magnesium alloy with electroless plating nickel interlayer. *Appl. Surf. Sci.* **2015**, *327*, 100–106, doi:10.1016/j.apsusc.2014.11.151.

181. Yan, P.; Ying, T.; Li, Y.; Li, D.; Cao, F.; Zeng, X.; Ding, W. A novel high corrosion-resistant polytetrafluoroethylene/carbon cloth/Ag coating on magnesium alloys as bipolar plates for lightweight proton exchange membrane fuel cells. *J. Power Sources* **2021**, *484*, 229231, doi:10.1016/j.jpowsour.2020.229231.
182. Yang, Y.; Guo, L.-J.; Liu, H. Corrosion characteristics of SS316L as bipolar plate material in PEMFC cathode environments with different acidities. *Int. J. Hydrogen Energy* **2011**, *36*, 1654–1663, doi:10.1016/j.ijhydene.2010.10.067.
183. Nikam, V.V.; Reddy, R.G.; Collins, S.R.; Williams, P.C.; Schiroky, G.H.; Henrich, G.W. Corrosion resistant low temperature carburized SS 316 as bipolar plate material for PEMFC application. *Electrochimica Acta* **2008**, *53*, 2743–2750, doi:10.1016/j.electacta.2007.10.061.
184. Jung, G.-B.; Chuang, K.-Y.; Jao, T.-C.; Yeh, C.-C.; Lin, C.-Y. Study of high voltage applied to the membrane electrode assemblies of proton exchange membrane fuel cells as an accelerated degradation technique. *Appl. Energy* **2012**, *100*, 81–86, doi:10.1016/j.apenergy.2012.06.046.
185. Papadias, D.D.; Ahluwalia, R.K.; Thomson, J.K.; Meyer, H.M.; Brady, M.P.; Wang, H.; Turner, J.A.; Mukundan, R.; Borup, R. Degradation of SS316L bipolar plates in simulated fuel cell environment: Corrosion rate, barrier film formation kinetics and contact resistance. *J. Power Sources* **2015**, *273*, 1237–1249, doi:10.1016/j.jpowsour.2014.02.053.
186. Le Bozec, N.; Compère, C.; L'Her, M.; Laouenan, A.; Costa, D.; Marcus, P. Influence of stainless steel surface treatment on the oxygen reduction reaction in seawater. *Corros. Sci.* **2001**, *43*, 765–786, doi:10.1016/s0010-938x(00)00113-x.
187. Park, Y.-C.; Lee, S.-H.; Kim, S.-K.; Lim, S.; Jung, D.-H.; Lee, D.-Y.; Choi, S.-Y.; Ji, H.; Peck, D.-H. Performance and long-term stability of Ti metal and stainless steels as a metal bipolar plate for a direct methanol fuel cell. *Int. J. Hydrogen Energy* **2010**, *35*, 4320–4328, doi:10.1016/j.ijhydene.2010.02.010.
188. Alishahi, M.; Mahboubi, F.; Khoie, S.M.; Aparicio, M.; Hübner, R.; Soldera, F.; Gago, R. Electrochemical behavior of nanocrystalline Ta/TaN multilayer on 316L stainless steel: Novel bipolar plates for proton exchange membrane fuel-cells. *J. Power Sources* **2016**, *322*, 1–9, doi:10.1016/j.jpowsour.2016.04.133.
189. Lee, S.; Woo, S.; Kakati, N.; Lee, Y.; Yoon, Y. Corrosion and electrical properties of carbon/ceramic multilayer coated on stainless steel bipolar plates. *Surf. Coatings Technol.* **2016**, *303*, 162–169, doi:10.1016/j.surfcoat.2016.03.072.
190. Mathabatha, M.; Popoola, A.; Oladijo, O. Residual stresses and corrosion performance of plasma sprayed zinc-based alloy coating on mild steel substrate. *Surf. Coatings Technol.* **2017**, *318*, 293–298, doi:10.1016/j.surfcoat.2016.10.023.
